# Oncoviruses: Induction of cancer development and metastasis by increasing anoikis resistance

**DOI:** 10.1016/j.heliyon.2023.e22598

**Published:** 2023-11-27

**Authors:** Zahra Sobhi Amjad, Ali Shojaeian, Javid Sadri Nahand, Mobina Bayat, Mohammad Taghizadieh, Mosayeb Rostamian, Farhad Babaei, Mohsen Moghoofei

**Affiliations:** a Department of Microbiology, School of Medicine, Kermanshah University of Medical Sciences, Kermanshah, Iran; b Research Center for Molecular Medicine, Hamadan University of Medical Sciences, Hamadan, Iran; c Infectious and Tropical Diseases Research Center, Tabriz University of Medical Sciences, Tabriz, Iran; d Department of Pathology, Faculty of Medicine, Tabriz University of Medical Sciences, Tabriz, Iran; e Nosocomial Infections Research Center, Kermanshah University of Medical Sciences, Kermanshah, Iran; f Infectious Diseases Research Center, Health Research Institute, Kermanshah University of Medical Sciences, Kermanshah, Iran

**Keywords:** Carcinogenic viruses, Anoikis, Cell death pathways, Metastasis, Cancer

## Abstract

The phenomenon of cell death is a vital aspect in the regulation of aberrant cells such as cancer cells. Anoikis is a kind of cell death that occurs when cells get separated from the extracellular matrix. Some cancer cells can inhibit anoikis in order to progress metastasis. One of the key variables that might be implicated in anoikis resistance (AR) is viral infections. The most important viruses involved in this process are Epstein-Barr virus, human papillomavirus, hepatitis B virus, human herpes virus 8, human T-cell lymphotropic virus type 1, and hepatitis C virus. A better understanding of how carcinogenic viruses suppress anoikis might be helpful in developing an effective treatment for virus-associated cancers. In the current study, we review the role of the mentioned viruses and their gene products in anoikis inhibition.

## 1 Introduction

**Fig. 1:**
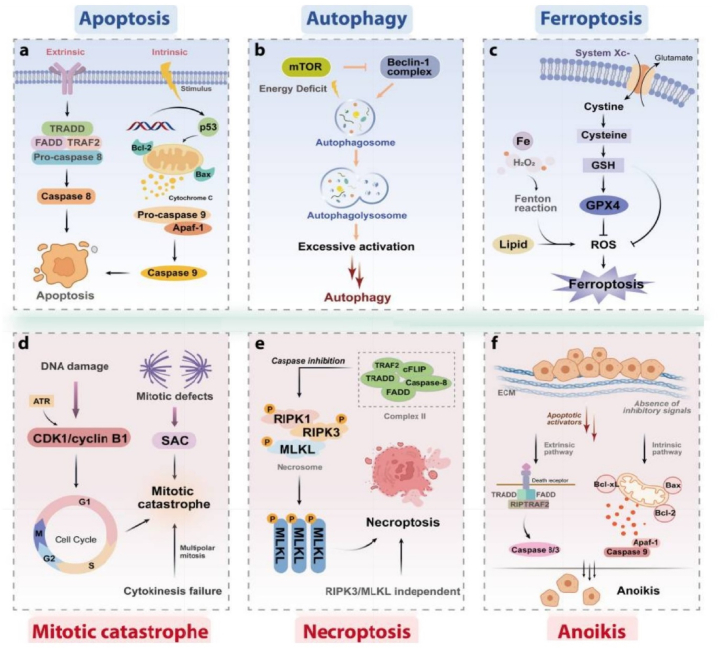
Molecular mechanism underlying autophagy, apoptosis, ferroptosis, anoikis, necroptosis, and mitotic catastrophe. a) The extrinsic and intrinsic pathways are two essential signal transduction cascades modulating cell apoptosis which are triggered via death receptors and ligands, and through mitochondria dysfunction mediated by cellular stress, respectively. b) Autophagosome formation requires mTOR regulation through modulating of Beclin-1complex, and also several proteins and signaling molecules such as PI3K, AMPK, and p62. c) Induction of ferroptosis occurs through the iron-dependent aggregation of lipid reactive oxygen species (ROS) and lipid peroxidation, the suppression of cystine/glutamate antiporter, and reduced glutathione peroxidase 4 (GPX4) activity. d) A critical component of mitotic catastrophe is the cyclin-dependent kinase 1 (CDK1)/cyclin B1 complex which is able to influence the transition of cell cycle from the G2 phase to the M phase. Number of critical factors resulting in mitotic catastrophes are DNA damage, cytokinesis failure, and mitotic defects. e) as a regulated necrotic cell death, necroptosis is promoted via tumor necrosis factor-α (TNF-α). After attachment of TNFα to the receptor, tumor necrosis factor receptor 1 (TNFR1) employs TNFRSF1A associated via death domain (TRADD), receptor-interacting serine/threonine kinase protein (RIPK) 1, Fas associated via death domain (FADD), TNF receptor-associated factor 2 (TRAF2) and other proteins to form complex I. Subsequently, deubiquitinating of RIPK1 occurs to facilitate the complex I conversion to complex II. In the situation of inhibition of caspase-8, mixed lineage kinase domain-like protein (MLKL), RIPK1, and RIPK3 are employed for necrosome formation by phosphorylating, which ultimately activates necroptosis. f) Though conservative apoptotic pathways, anoikis promote cell death. B cell lymphoma 2 (Bcl-2) related proteins are implicated in the modulation of anoikis, and several protein kinases contribute to anoikis signal transduction. When the detachment of cells from the extracellular matrix (ECM) occurs, activation of pro-survival signals gets interrupted. Meanwhile in order to avoid adherent-independent cell growth and attachment, triggering of the death receptors as well as mitochondrial apoptotic pathways occurs, which eventually leads to anoikis activation to promote cell death [73].

The phenomenon of cell death is one of the processes that control and regulate the abnormal growth of cancer cells or prevent the cancer cells from spreading and invading different sites within the body. Cell death is crucial in regulating the body's natural functioning and various pathological states. For ease of understanding, cell death is generally divided into purposeful death (apoptosis) and unintentional death (necrosis). However, one classification lists 11 different kinds of cell death, namely, autophagy, apoptosis, ferroptosis, necroptosis, mitotic catastrophe, and anoikis (Fig. 1) [1,2]. Initially described by Frisch and Francis in 1994, anoikis refers to the fading of the correct connection between the cell and extracellular matrix (ECM). In fact, anoikis could be a kind of programmed cell death derived from the Greek word meaning “home loss” in epithelial and endothelial cells [3,4]. This phenomenon is important for tissue homeostasis and appropriate cell growth control [5]. Cells fall and perish when removed from the extracellular matrix (ECM) due to a lack of cellular adhesion molecules such as integrins. On the other hand, linking to unrelated sites by integrins leads to the loss of signals required for survival and growth. Integrins and some growth receptors exchange growth signals within the ECM, such as epidermal growth receptors [2,[6], [7], [8]]. Cancer cells enhance angiogenesis and metastasis to non-involved organs when this form of cell death is inhibited [9]. By modifying biological mechanisms, tumor cells become resistant to anoikis [[10], [11], [12]]. However, it's still unclear what causes anoikis resistance (AR) and cancer cell metastasis.

Viruses cause about 12% of global cancer occurrences, with the great majority of these cases taking place in developing countries. Epstein-Bar Virus (EBV) and Human Papilloma Virus (HPV) are linked to 38% of all malignancies caused by viruses [13]. In addition to EBV and HPV, Hepatitis B virus (HBV), Hepatitis C virus (HCV), Merkel cell polyomavirus (MCPyV), Kaposi's sarcoma virus (KSHV), and human type 1 L lymphotropic virus (HTLV-1) are five viruses with the cancer-causing potential in humans [14,15]. Some of the proteins found in these viruses decrease tumor suppressor signaling, prevent cell death pathways, increase AR, promote metastasis and angiogenesis, and contribute to the transmission of viral infections and cancer [13]. The inhibition of anoikis of cells isolated from the matrix is one of the mechanisms that contribute to metastasis progression, and several studies have shown that viral proteins interfere with the regulatory pathways of anoikis and planned apoptosis to the progression of malignancy [4,16,17].

Anoikis is the process of eliminating detached or misplaced cells under pathological or physiological situations, which facilitates tissue homeostasis [18,19]. Overall, anoikis refers to a subset of the apoptosis mechanisms, which are classified as intrinsic and extrinsic pathways [19]. Metastasis in cancer cells gets retarded to other parts by anoikis; though, this is mostly not due to the anoikis-resistance of cancer cells. Nevertheless, a comprehensive understanding of the mechanisms, underlying AR in cancerous cells is a must [20]. Consequently, achieving a general understanding of the AR mechanism might aid in evading tumor progression and metastasis. Several studies reported that some viral infections can serve a crucial role in the regulation of anoikis pathways [4,[21], [22], [23], [24], [25]]. Reportedly, resistance to anoikis-mediated cell death has been established in a few virus-infected cells [26,27]. For example, through suppression of tumor suppressor genes, anchorage-independent high-risk HPV (hrHPV)-transformed cells evade anoikis [28]. Also, viral products (proteins and/or non-coding RNAs) can control the expression of anoikis-participating factors by targeting essential factors implicated in the regulation of either death receptors (extrinsic pathway) and/or mitochondria (intrinsic pathway). For example, some viral proteins can interact with the Bcl-2 family [[29], [30], [31], [32]], TNF superfamily members [33], caspase-8 [[34], [35], [36], [37]], ECM [[38], [39], [40]], integrin [41], and E-cadherin [[42], [43], [44]] as well as viruses can encode viral analogs of the Bcl2 protein [32] that functions in the regulation of anoikis are still unknown. Hence, viral infections may potentially modulate cancer cell behavior by affecting the initiation and/or development of AR which, in turn, promotes tumorigenesis. Here, we will review the existing data on the regulatory role of viral infection in AR which highlighted controlling viral infection as an alternative approach for targeting AR in cancerous cells. This study focuses on the mechanism by which carcinogenic viruses contribute to cancer development and metastasis by regulating the anoikis pathway. It is hoped that a more comprehensive understanding of how carcinogenic viruses control anoikis will aid in developing effective therapies to treat virus-related malignancies.

## 2 Anoikis

Cell-ECM interactions are critical for cell survival and growth. The ECM is composed of molecules proteoglycans, elastic fibers, adhesion receptors cadherins, selectins, and integrins [45]. The ECM acts as a scaffold that provides the necessary signals during cell growth and differentiation [46]. Loss of adhesion between cells and ECM leads to cell dysfunction and various diseases including cancer [47,48]. Induction of apoptosis in cells with loss of adhesion and detached cells from the extracellular matrix or adjacent cells causes a special form of apoptosis called anoikis, which was initially defined in 1993 by Meredith et al. [49,50]. Anoikis (“homelessness”) is crucial for homeostatic control, signaling for endothelial cell survival, and tumor suppression [51,52]. The initiation of anoikis as one of the essential processes for homeostasis, cell survival, and tumor suppression, is facilitated by the intrinsic and extrinsic caspase activation comparable to the activation of apoptotic cascade of endonucleases, DNA damage, and cell death [53]. Integrins are responsible for the attachment of cells to the extracellular matrix [54]. Changes in molecules required for cell-cell adhesion, integrins, integrin-associated signaling molecules, or apoptotic regulators in malignant cells can cause AR, which makes it easier for the cells to separate from the primary lesion, promote anchorage-independent growth, stay in the body, and spread the metastasis-causing seeds [55,56]. Cancer cell AR is a phenomenon that is becoming more well-known as cancer cells continue to alter their strategy of distant metastasis [57]. Thus tumor cells need the AR, for proliferation and attacking the normal epithelial cells, leading to the extension of cancer [50]. Integrins govern (regulate) this phenomenon by forming adhesion complexes with the ECM, while the actin cytoskeleton creates (forms) cell protrusions that attach to the ECM and guide cell migration [51,58]. Integrins are heterodimeric transmembrane receptors that trigger the cell signaling pathway for cell-ECM transition [59]. Therefore, Integrin receptors mediate the interaction of cell-ECM and provide physical attachments to the cytoskeleton. Besides, they also participate in the signal transition from the ECM to the cells that are implicated in a variety of histological, hemostatic, developmental, and oncogenic procedures [18]. Activation of early integrin signaling involves invoking cytoplasmic protein kinases including SRC family kinases (SFKs), focal adhesion kinase (FAK), and integrin-associated kinase (ILK), which have been associated with cell survival [55,60,61]. The modulation of the pro-apoptotic Fas-associated death domain protein (FADD)/Caspase-8 and jun kinase, as well as the PKB/Akt survival pathways, are confirmed to be connected to the suppression of anoikis [62].

### 2.1 Molecular pathway of anoikis

Anoikis is regulated by the interaction of two apoptotic pathways, i.e., mitochondrial disruption and activation of cell surface death receptors (ADCC), which in turn activates caspases, and subsequently activates endonucleases and DNA fragmentation, ultimately leading to cell death [19,63,64]. Bcl-2 family proteins affect extrinsic and intrinsic pathways of apoptosis. The three families of the Bcl-2 proteins regulating the mitochondrial outer membrane permeabilization (MOMP) a crucial stage in the intrinsic apoptosis process, contain members that either promote or prevent apoptosis [65].

1. Anti-apoptotic proteins, such as Bcl-2, BFL-1/BCL-2A1, BCL-W, Bcl-XL, and Mcl-1.
2. Proapoptotic proteins, including Bax, Bak, and Bok.
3. BH3 multi-dominant proteins, such as Bad, Bim, Bmf, Bid, Bik, Noxa, and Puma [66,67].

Integrins mediate FAK and ILK phosphorylation which in turn trigger the PI3K/AKT, ERK/MAPK, and JNK signaling pathways. Consequently, integrins mediate resistance to either extrinsic and intrinsic apoptosis or influence the tumor progression and cell survival through elevating NF-κB, Jun, and Fos transcription. The involvement of integrin is also able to repress Fas death receptor expression. This transcription results in adhesion-dependent survival and AR in cancerous cells via mechanisms that require activating mesenchymal-epithelial transition (EMT) and downregulation of redox-mediated proapoptotic factors [18,68,69].

#### 2.1.1 The intrinsic pathway

The intrinsic route is triggered by multiple intracellular signals, including DNA damage and endoplasmic reticulum stress, and mitochondria take an important part in the regulation and control of apoptosis. The pro-apoptotic Bax/Bak proteins travel from the cytosol to the outer mitochondrial membrane (OMM) in response to death signals, inducing mitochondrial permeability and cytochrome C release. Cytochrome C secretion causes apoptosomes to develop, including cytochrome C, Apaf, and caspase 9, which then activate caspase-3 and carry out the proteolysis procedure. Fig. 1 depicts the mechanism of programmed cell death [18].

#### 2.1.2 The extrinsic pathway

For apoptosis induction, extracellular death ligands are recruited by the extrinsic apoptosis pathway. For instance, FAS/CD95, TRAIL, TNFR1 DR4, and DR5 are a number of such ligands [70].

Extracellular death ligands like Fas L or TNF- bind to membrane receptors like Fas and TNFR to activate the external route. Caspase 8 is then triggered by forming a lethal signaling complex (DISC) with FADD, which starts caspase-3 proteolytically, resulting in substrate proteolysis and cell death [71].

The binding of TNF, FasL, or TRAIL-related ligands to death receptors (DRS) such as TNFR1, Fas, or DR4 activates the external apoptotic pathway. This interaction leads to the tetramerization of adapter proteins including the second TNF-1 receptor-dependent death and FADD, followed by other cellular factors in the cytoplasm binding to the second intracytoplasmic receptor of these receptors and, in turn, activating caspase 8 is the precursor of procaspase 8. In the lack of cellular IAPs (c-IAPs), stimulation of the TNFR1 receptor precludes the receptor 1 cross-protein (RIP1) ubiquitinating, which in turn allows RIP1 to connect to FADD and caspase-8 to create a proapoptotic cytoplasmic complex. Interaction of non-ubiquitinated RIP1 with FADD and RIP3 results in apoptosis-independent necroptosis. The innate apoptosis pathway can result from cytotoxic agents and includes releasing the mitochondrial content into the cytosol, for instance, cytochrome *c* and SMAC. Mitochondrial permeability can also be caused by the caspase-8 conversion of the caspase-induced cross-death domain agonist BH3 (BID) to short BID (tBID). Through binding and degrading several IAPs, and cytochrome *c* activates caspase-9, SMAC is able to promote apoptosis. X-linked IAP (XIAP) serves as a direct antagonist of caspase-3, -7, and -9 [72].

#### 2.1.3 Healthy cells are immune to anoikis

As mentioned above, anoikis refers to apoptosis that is induced by detachment of cell from the ECM [74]. Adhesion to the ECM is required for survival and the proliferation of normal epithelial cells require, and reduction of this adhesion leads to anoikis. However, anoikis provides protection for epithelial cells in multiple conditions, such as constant ECM contacts or momentary release from the ECM occurring in both mobile and dividing cells. Consequently, mesenchymal cells often have elevated AR as a result of their high mobility [75,76]. Additionally, professionally non-adherent cells, including mature hematopoietic cells and leukocytes, exhibit protection against anoikis [77].

Mainly, adherence to permissive ECM proteins prevents cells to initiate anoikis. Therefore, the implication of ECM in suppressing anoikis is very well determined [78,79]. Furthermore, multiple integrins (α1β1, α2β1, α3β1, α5β1, α6β1, α6β4, αvβ3) dispose a great influence on the survival [[80], [81], [82]] of neoplastic or normal cells [[82], [83], [84]]. Critical contributors to signal transduction mediated by integrins which leads to anoikis protection are FAK, Src tyrosine kinase, integrin-linked kinase (ILK), ERK, PI3K, and the adaptor protein Shc. Through integrin ligation by proper ECM proteins, ILK and FAK are employed and trigger ERK, PI3K/Akt, and the Jun-kinase (JNK) pathway [85,86]. Activated PKB/Akt suppress several levels of the anoikis program, including deactivation of caspase-9 and pro-apoptotic protein Bad phosphorylation, stimulation of NF-κB, along with reduction of Fork-head transcription factors [[87], [88], [89], [90]]. ILK transfer integrin-mediated survival signals in a FAK independent manner, as implied via failing of dominant-negative FAK to inverse to the protection mediated by ILK against anoikis [62,91]. Moreover, FAK or ILK independent transduction of adhesion signals to ERKs could also occur via the adaptor protein Shc [92,93]. Bim phosphorylation and its subsequent inactivation is due to negative regulation of ERKs and PI3K. Bim phosphorylation results in degradation of BH3-only protein and subsequent cease of Bim from antagonizing Bcl-2 function or accelerating the activation of Bax. Consequently this results in anoikis blockage [74]. A practical cross-talk among integrins ligated to ECM proteins and numerous growth factor receptors could be developed by adherent cells. There are several growth factor receptors employed in integrin platforms [74] such as, platelet-derived growth factor receptor (PDGFR), epidermal growth factor receptor (EGFR), vascular endothelial growth factor receptor (VEGFR), and hepatocyte growth factor receptor (HGFR) [[94], [95], [96], [97]]. Stimulation of ERK and PI3K signaling in epithelial cells, occurs through activation of either integrin-mediated ligand-independent or ligand-dependent EGFR, which results in inhibition of the apoptotic factors activity [74]. Disengaged β1 integrins in detached cells, results in inhibition of EGFR expression concluding in elevation of Bim death signaling [98]. Expression of EGFR is then inhibited via prolonged suspension, thus resulting in reduction of its survival signaling. On the other hand, the restoration of ECM contact enhances the EGFR expression and its pro-survival spur [74]. EGF receptor phosphorylation in a manner independent of ligand in response to ligation of integrin is actually relied on its correlation with the adaptor protein p130Cas and the c-Src tyrosine kinase [74,97,99]. Activation of Src kinase occurs in result of integrin ligation in consequence of its redox sensitivity. Small GTPase Rac-1 activation and reactive oxygen species (ROS) production is derived by engagement of integrin. Consequently, generated ROS oxidize and triggers Src kinase, which contributes to EGFR *trans*-phosphorylation independent of ligand. For this purpose, active Src is invoked at the integrin/EGFR platform, and switches both ERK and PKB–Akt pathways [74]. Bim degradation and anoikis evading are the final occurring events. Eventually, autophagy is coordinated with AR of epithelial cells [74,[100], [101], [102]]. In fact, ECM-detached cells survival is facilitated by the RNA-activated protein kinase-like endoplasmic reticulum kinase (PERK), meanwhile enhancing autophagy and the production of ATP. ECM detachment triggers autophagic pathway via ATG6 and ATG8, maintains ATP levels, and interrupts anoikis. Beclin-1, critical component in such integration, is an autophagic protein participating in the modulation of the anti-apoptotic role exerted by Bcl-2 and Bcl-XL, ROS, and ERKs [74,103,104]. Survival of epithelial cells is offered by autophagy due to their re-adherence onto the ECM in a time-consuming manner. Therefore, same settings that have been utilized by circulating cancerous cells to avoid anoikis, is involved in facilitation of the tumor cell dormancy via nutrient recovery, and metastases distribution [74,105].

#### 2.1.4 Anoikis resistance in cancer cells

Anoikis, an apoptotic process that normally occurs in healthy cells, stops metastasis. This process prevents cell proliferation in the incorrect locations, which is especially important during the metastatic phase, and is essential for tissue homeostasis. After separating from the original sites, cancer cells move forward to metastasis to survive by becoming resistant to anoikis. AR promotes the ability of cancer cells to invasion and spread within the body, leading to metastasis [106,107]. So, adhesion molecules in stabilizing ECM take an essential part for anoikis, and alteration of them is able to grant AR, which is required for metastasis [68].

The physiological process of EMT enables epithelial cells to modify the cytoskeleton, release the linkage with vicinal cells and obtain a motile phenotype. Activation of this phenomenon often occurs during inflammation, wound healing, or embryogenesis. In cancerous cells EMT is confirmed to participate in detachment from adjacent cells, prevent anoikis, localizing from their initial location, and invading other tissues [108,109]. EMT is a multifaceted, complex condition that frequently comprises episodic, transitory, or partial occurrences. Transcription factors including Twist, Snail, and ZEB1/2 are able to trigger the phenotypic EMT. Activation of Twist participates in EMT by accelerating invasion and migration, and elevates the antiapoptotic protein Bcl-2 to trigger AR [110,111]. As E-cadherin repressor, Transcriptional activation of Snail-1 participates in the induction of EMT [112]. Besides Snail is also capable of suppressing the transcription of genes implicated in anoikis, including PTEN. Decreased PTEN triggers the PI3-K/Akt pathway and deactivates the proapoptotic protein Bad, which participates in AR. Furthermore, during EMT downregulation of E-cadherin accelerates the cytosolic aggregation of free β-catenin and further nuclear translocation. Subsequently, induction of genes participating in the modulation of cell invasion, migration, and cell cycle progression, including c-Myc, cyclin D1, c-Jun, and MMP-1 occurs [113,114]. Upregulation and accumulation of β-catenin in the cytosol results in AR by regulating c-Myc, cyclin D1, and MAPK-mediated survival pathways and suppression of epithelial genes toward maintaining a mesenchymal phenotype among cancer cell populations [115]. EMT is accelerated by the ZEB1 transcription factor [[116], [117], [118]] through elevating vimentin and reducing E-cadherin and semaphorin 3F, leading to triggering Akt survival pathway and AR [116,119].

ECM generates several integrated stressors, such as glucose absorption, ATP limitation, oxidative stress, and a significant increase in ROS in mitochondria, as the initial stage of metastasis [120]. The oxidative stress is suggested via ROS generation in tumor cells. In gastric and lung cancer cells, ROS production due to the upregulated NADPH oxidase 4 (NOX4) was confirmed to promote AR and metastasis through EGFR signaling. Additionally, enhanced ROS have been established in cancer stem cells (CSCs) as a metastatic trait to elevate EMT and the invasiveness of multiple cancer cell lines [19]. In contrast, in an AR glioblastoma cells with elevated expression of hypoxia-inducible factor (HIF), a low ROS content has been observed [46]. Similarly, the upregulated manganese superoxide dismutase elevates AR and tumor metastasis, suggesting that the ROS elevation leads to increasing anoikis sensitivity of the cells upon their detachment [47,48]. Despite all the efforts, more investigations are required for the determination of ROS role and level in cancer cells and the tumor environment.

Glycolysis is the process of glucose oxidation to pyruvate commencing with hexokinase phosphorylating a glucose to provide glucose-g-phosphate (G6P). In limited oxygen conditions, lactate dehydrogenase converts pyruvate to lactate to provide the required energy needs [160]. 36 ATPs per molecule of G6P are the result of oxidative phosphorylation (OXPHOS). Whereas the G6P-lactate pathway provides two ATP molecules, it happens spontaneously and efficiently, consequently allowing both paths to provide enough mount of ATP required for cancer cell growth and proliferation [68]. Accumulated ATP by the G6P-lactate reactions is utilized via cancer cells for their proliferative machinery, and enables them to evade ROS generation from the OXPHOS path. OXPHOS path is disadvantageous for dysregulation of various signaling pathways culminating in acidosis of the tumor environment, AR, uncontrolled cell growth, migration, and metastasis [52,121]. It has been established that highly invasive TNBC variant show metabolic perturbation due to using glucose via the glycolytic path to cover its energy need in contrast to its less invasive variant; targeting both variants with 2-deoxy-glucose reduced glycolysis and triggered anoikis sensitivity of more aggressive cell lines [122].

Cancer cells with reduced glycolysis are able to survive, proliferate, and metastasize. It has been established that cyclic adenosine protein kinase A (PKA) is able to confer anoikis resistance in cancer cells under glucose limited condition [123]. In AR cells the hyperactivation of PKA induced autophagy and glutamine metabolism to provide the required energy. Reduced cancer cell survival and anoikis sensitivity of the cells is due to the blockade of PKA activity [123]. It previously has been confirmed that the growth of epithelial human breast cancer cell line, MDA-MB-231 cells, in anchorage-independent circumstances leads to elevated production of ATP via fatty acid oxidation (FAO). It has been revealed that the accumulated glucose in the cell is utilized to fuel PPP for redox homeostasis. The elevated function of PPP further promotes AR. The MDA-MB-231 cells treatment with a synthesized flavonoid (GL-V9) results in elevated AMPK-activated lipid oxidation, production of ROS, limited PPP, and enhanced anoikis sensitivity [68]. Furthermore, it has been confirmed that serum and glucocorticoid kinase-1 (SGK-1) are implicated in accessibility of energy for cells detached from ECM. Therefore, this elevated glucose uptake through upregulation of GLUT1 expression and PPP for enhanced ATP production enables AR and survival [124].

Apoptosis is also suppressed by EGFR receptor stimulation and subsequent triggering of the ERK and PI3K/Akt signaling pathways. ERK and Akt's activation leads to Bim phosphorylation (Bim protein stimulates apoptosis in the mitochondrial pathway by interacting with Bcl-xL and Bcl-2 proteins) and thus degrades it. Conversely, loss of integrin-binding dramatically increases Bim expression and cell death. Therefore, integrins (cell adhesion) and EGFR signaling may regulate anoikis via modulating Bim cytoplasmic expression [107]. Increased N-cadherin expression leads to persistent Akt activation. According to studies, switching from E to N-cadherin frequently occurs in cancer epithelial cells undergoing EMT [125]. According to research on the PI3K/Akt signaling pathway in AR endothelial cells, PI3K activates Akt by absorbing N cadherin. Activating the PI3K/Akt signaling pathway is the most common means for cancer cells to develop AR, and PTEN is the most important regulator of this system [18,126]. AR is more substantial in metastatic cells of the large intestine than in normal intestinal epithelial cells. These anoikis-resistant cancer cells had higher levels of integrin-α2, β1, and EGFR-activated in the anchor-independent state than in the anchor-dependent state. On the other hand, normal intestinal epithelial cells are incapable of increasing the level of expression of these proteins. Thus, in the lack of ECM, EGFR-associated integrins results in activation of ERK and AKT and induce AR via inhibiting caspase-3 activation [127].

## 3 Human oncoviruses

Infective factors including viruses, bacteria, and other parasites are associated to approximately a fifth of carcinogenesis. Viral infection is annually accounting for 19% and 4% of new cancer cases in developing and developed countries, respectively [128]. A seventh of 1400 human pathogens that have been described are viruses among them, oncogenic viruses considered a threat due to their cancer induction ability [129]. HBV, HCV, HPV, EBV, human herpes virus 8 (HHV8), HTLV-1, and MCPyV are among important human oncogenic viruses. HBV or HCV infection account for 80% of hepatocellular carcinoma (HCC) cases. Cervix cancer, and some head and neck tumor are associated with HPV infection. Responsible for epithelial cells and lymphocytes infections, EBV causes Burkitt’s lymphoma, Nasopharyngeal carcinoma (NPC), and Hodgkin’s lymphoma [130].

HHV-8 is correlated with Kaposi’s sarcoma (KS) and various hematologic malignancies. The risk of various human malignancies is higher for patients with HHV8 infections and a background of lymphoproliferative diseases and chronic blood [131]. In addition, MCPyV (skin normal flora) is responsible for Merkel cell carcinoma (MCC) and HTLV-1 for adult T-cell lymphoma/leukemia [132]. The only carcinogenic polyomavirus, MCPyV, is responsible for the development of MCC [133]. While polyomaviruses are not considered lethal for healthy individuals, they can commence disastrous diseases in immunocompromised subjects [134].

Each virus accounts for a different amount of new cancer cases worldwide. Besides, developing countries account for at least 85% of virus-associated cancers, which suggests healthcare interventions be urge at the public level [135].

The oncogenic viruses are divers in term of proportion and cancer type that they induce, based on the geographical region. In that regard, the prevalence of oropharyngeal cancer in result of HPVs infection, depends on the geographical area and is constantly rising, specifically in industrialized countries such as the US. Additionally, HPV is considered as a factor for almost half of the vulvar and nearly entire cervical and other anogenital cancers [135,136].

### 3.1 The role of the virus in regulating anoikis

Carcinogenic viruses or oncoviruses, including the HBV, HCV, HPV, EBV, MCPyV, and HHV8 or Kaposi's sarcoma-associated herpesvirus (KSH) account for 12% of all cancers in humans [14,15]. Oncoproteins in these viruses alter cell behavior by suppressing the tumor suppressor signal pathway, preventing cell death pathways, increasing AR, driving metastasis and angiogenesis, and promoting infections of aids in the progression of viral neoplastic diseases [13].

#### 3.1.1 HBV

As a member of the hepadnaviridae family, HBV has a double-stranded (ds) DNA that is approximately 3200 bp long and 52–48 nm in diameter, consisting of a nucleocapsid nucleus enclosed in a glycolipid envelope [137,138]. HCC, or liver cell cancer, is caused by HBV, specifically the protein X virus (HBx). Studies have revealed that AR could get spread in HCC [139]. HBV X protein, also known as HBx, is a 17-kDa carcinogenic protein encoded by the HBV genome that serves an important role in developing HBV-related HCC. By interacting with transcription factors, HBx can affect viral and cellular promoters and serve a crucial role in the pathogenesis of HCC via regulating the cell cycle, proliferation, and apoptosis [140,141]. The possible role of HBV and HBx oncoprotein in suppressing anoikis is discussed in this section. In hepatoma and hepatic cells, HBx expression has been shown to promote epithelial-mesenchymal/AKT/transmission and activate the snail protein via activating the PI3K/AKT/GSK-3b signal pathway, which aids EMT and the spread of hepatoma attack in vivo and in vitro. The HBx oncoprotein changes E-cadherin to N-cadherin, ultimately activating the PI3K/AKT signaling pathway and inhibiting anoikis. Furthermore, HBx induces EMT via activating the twist and snail promoters by increasing the STAT3 and PI3-K/Akt/GSK-3b signaling cascades [4,142]. Reportedly, HBV-HBx protein can contribute to the development of HCC metastasis by increasing the expression level of factors promoting metastasis such as DDX17, and RHAMM [143,144]. Besides, HBV-HBx protein aggravates metastasis by activating the AKT-NFκB signaling pathway through regulating miR-520c-3p/PTEN [145]. HBx protein negatively regulates the anti-metastatic factors SHIP2/SKP2 [146] and GNA14 [147] as a result leads to the induction of cancer metastasis. HBx, which is required to influence transcription factors such as AP-1 and NF-kB, triggers the MAPK signal cascade, including P38/MAPK, PI-3K, and JNK, started by HBx. The activation of the ERK and PI-3K-AKT/PKB pathways boosted AP-1 and NF-B transcriptional activity in HBx-transfected hepatocytes, leading to higher MMP-9 production and cell survival. HCC invasion and metastasis depend on MMP-9 matrix metalloproteinase expression. HBx has also been shown to induce liver cancer by suppressing the expression of PTEN, a tumor-suppressing gene [148,149]. Furthermore, the HBx protein can inhibit mitochondrial enzymes involved in electron transfer for oxidative phosphorylation, resulting in more oxygen-free radical species, which can damage mitochondria or induce cell death [141]. Fibulins are a group of ECM proteins with the ability of modulating cell proliferation and migration. Fibulin-1 binds to some ECM proteins, including fibronectin and laminin-1, and regulates cell morphology, stimulation, and proliferation via signal transduction. Fibulin-1 transcription results in the expression of four types of fibrins called fibulin 1A to fibulin 1D, which differ only at the C-terminus. In fibrosarcoma-derived cells, increased fibulin-1D expression inhibits anchor-independent proliferation. It has been observed that the mean Fibulin-1 expression is higher in ovarian and breast tumors than in other malignancies. Fibulin-1 also prevents apoptosis via the Notch pathway and can target the Mcl-1 and Bcl-xL genes. Fibulin expression is higher in HBV-infected hepatocytes, and hypermethylation of the fibulin-1 promoter inhibits tumor growth in HCC. Fibulin-1 silencing makes HCC cells more sensitive to apoptotic signals and lowers their potential to form tumors in vivo [150]. According to research, HBV virus proteins, particularly the HBx protein, can help the progression of liver cell cancer by influencing the pathways involved in anoikis. Table 1 provides further details.

**Table 1:** Carcinogenic proteins of viruses involved in inhibition of anoikis.

Virus	Virus protein	Target	Mechanism of resistance to anoikis	Reference
HBV	HBx	EMT	Activation of the snail protein	[4,142]
	HBx	E-cadherin, N-cadherin	Anoikis control and activation of the PI3K/AKT signaling pathway	[4,203]
	HBx	Twist	Snail activation and EMT induction	[4,188]
	HBx	MAPK	Transcript factors like AP-A and NF-kB should be activated	[148,149]
	HBx	PI-3K-Akt/PKB, ERK 1/2	Increase cell survival	[204]
	HBx	PTEN	Liver cancer progression	[148,149]
	HBx	MMP9	Increased HCC metastasis	[148,149]
	HBx	Mitochondrial enzymes	Mitochondrial damage and increased cell death	[141]
	Fibulins	Laminin 1 and fibronectin	Stimulation and proliferation of cells	[150]
	Fibulins D1	E6	Alteration and inhibition of E6 protein	[150]
	Fibulins	MCL-XL and MCL-1	Inhibition of apoptosis	[150]
EBV	LMP-1	TrkB/NTRK2	Increased NPC cells against amplification, migration, and invasion of NPC cells	[155]
	LMP-1	ERK/MAPK	Induction EMT	[154]
	LMP-1	miR_ BART	Modulation of NF-κB activation leads to increased BART levels in NPCs	[157]
	LMP-2A	BARTs, EBERs, and LMP-1/2A	Increased resistance to anoikis	[152]
	LMP-2A	NF-kB	Increased anti-apoptotic signaling and ultimately inhibited apoptosis	[205,206]
KSHV	vFLIP	MVEC	Activation of the classic NFKB pathway and the RelA/p65 nuclear transfer	[207]
	vFLIP	COX2/PGE2	p38, Rac1-GTPase, RSK, FAK, Src, and Akt	[174]
	vFLIP	Secretion of cytokines	–	[207]
	Vfli/K13	COX2/PGE2	Effect on NF-KB and induction of tumorigenesis	[174]
	VGPCR	FAK	Activation of AP1, NF-KB, ERK1/2, and inhibition of anoikis	[175,208]
	VGPCR	PI3K/Akt	EC survival	[208]
	VGPCR	SAPK/MAPK	Activation of transcription factors such as AP1, CREB, NFAT, and HIF1a	[175,208]
HPV	E7	P600	Promote anoikis escape	[177]
	E6/E7	Notch	PKA/AKT activation and anoikis resistance	[182]
	E6/E7	EMT	E-cadherin expression is reduced by increased expression of EMT transcription factors such as SLUG, TWIST, ZEB1/2, and SIX1.	[208]
	E6/E7	miR-125	Increased metastasis and tumor spread	[186]
	E6/E7	E-cadherin N-cadherin	EMT is caused by a decrease in E-cadherin and an increase in N-cadherin	[208]

#### 3.1.2 EBV

EBV, or human gamma herpes virus, is a herpes virus with a genome of 184-kb linear dsDNA enclosed in nucleocapsid with 20 sides and a viral envelope capable of encoding approximately 100 viral proteins. EBV, the first human virus to be directly involved in carcinogenesis, is the cause of Burkitt's lymphoma, Hodgkin's disease, non-lymphoma, Hodgkin's NPC, lymphoma, and leiomyosarcoma in immunocompromised individuals. The virus is also associated with epithelial malignancies in the stomach and breast area. Humans act as the only natural hosts for EBV, despite the fact that herpes viruses are epidemic in nature [150,151]. According to studies, the ERK pathway is fundamentally activated in most EBV-positive cells. AR is reduced when ERK activity is inhibited. Therefore, EBV-positive cells are more withstand to anoikis than EBV-negative cells. LMP2A is one of the viral genes that cause ERK-mediated AR in EBV-positive cells [152]. It has been reported that EBV-encoded proteins (EBNA1, BARTs, EBERs, and LMP-1/2A) can play a crucial role in cancer AR in gastric cancer (GC) and NPC cells that have been infected with EBV [152,153]. LMP-1 is one of the oncoproteins of the EBV virus and can stimulate various carcinogenic pathways and acts as a TNF receptor in several signaling pathways, including NF-B1 and ERK-MAPK, NF-kB2, PI3K/Akt, p38-MAPK, JNK/SAPK, and TGF- which are regulated in various cancers. LMP1 also induces EMT with its CTAR1 domain via integrin ERK-MAPK signaling [153,154]. LMP-1 can improve AR in NPC cells by increasing the NTRK2 expression or TrkB via NF-kβsignaling and their motility and invasion [155].

LMP 1 is able to suppress and inhibit anoikis in two ways: i) by elevating the expression of anti-apoptotic proteins including survivin and CD44. and ii) by conferring resistance to the growth inhibitory cytokine TGF-β. By targeting the NF kB pathway, LMP 2A can increase anti-apoptotic signaling activity, leading to survivin production and ultimately inhibiting and blocking apoptosis. According to research, the EBV genome's two tiny non-coded RNAs, EBER1 and EBER2, encode approximately 180 nucleotides. EBERs are transcribed by host RNA polymerase III and contribute to immune suppression and apoptosis resistance by stimulating IL-10 and increasing Bcl-2 expression, respectively, in carcinogenesis [156].

EBVs have also been discovered to encode EBV BART, also known as viral miR or vmiRNA, a type of miR, with an unknown functioned. However, in EBV-positive gastric cancer and EBV-infected B cells, BART RNAs and viral miRs are both abundantly expressed in NPCs. Still, they are less so in EBV-positive GC and EBV-infected B cells. BART miRs boost BART levels in NPC cells by regulating NF-kβ activation via LMP-1(157). Mostafaei et al. stated that EBV-positive breast cancer cells had much greater levels of TGF-expression than EBV-negative breast cancer cells [158]. In EPV-infected NPC tissue lysis, the expression level of TBF-1 was also significantly elevated in contrast to non-malignant nasopharyngeal mucosa [159].

Recently, some studies have established that EBV serve a critical role in EBV-related cancer metastasis as well a significant correlation has been demonstrated between the presence of EBV with metastasis. For example, EBV-LMP1 protein can increase Ca^2+^ levels by acting on STIM1 and ultimately leads to the induction of NPC metastasis [160]. In addition, EBV-LMP1 promotes metastasis in NPC by downregulating E-cadherin expression and inducing EMT through activating NF-kB, PI3K/Akt, and MAPK signaling pathways [161,162]. Besides, EBV-encoded microRNAs such as miR-BART8-3p, miR-BART22, and BART2-5p can trigger cancer metastasis by regulating NF-κB and Erk1/2 pathway [163], Wnt/β-catenin signaling pathway [164], and Rho signaling pathway [165], respectively. It has been found that TGF-1 expression and secretion have also been induced by EBNA1 and LMP1 in epithelial cells in vitro [4,166]. The CD44 protein is another biological component implicated in metastasis. It is a multifunctional cell membrane receptor implicated in cell adhesion, tumor invasion, and metastasis in many malignancies, including prostate cancer. Anti-apoptotic proteins, CD44, DNA 1-binding inhibitor (Id1), Bim, and ROS have all been linked to EBV-LMP1-induced AR [49,167]. Also, co-expression of LMP1 causes cell proliferation, apoptosis resistance, anchorage-independent growth, and tumor formation in mice [168]. Mostafaei et al. reported that the mean CD44 expression in EBV-infected breast cancer groups rose considerably compared to healthy breast cancer groups; however, the level of PTPN expression did not change significantly [158]. PD-1 as a glycoprotein that alters the CTL surface cells and leads to the fatigue or exhaustion of T lymphocytes is expressed by cancer cells and stromal immune cells in the tumor. Cancer cells escape the reach of killer T lymphocytes due to this occurrence, which contributes to the development of cancer. According to studies, EBVaGC has been shown to contribute to the advancement of cancer by overexpressing genes such as PD-L1 (also known as CD274) and enhancing lymphocytosis in the tumor environment. Permeable tumor CTLs are inhibited and prevented from invading tumor cells when PD-L1 and PD-1 interact [169,170]. EBV may be able to evade the immune system by employing encrypted arrays. According to studies by Ion et al. [168]. PIAS3 is directly targeted by miR-BART5-5p which enhances PD-L1 expression via modulating the miR-BART5/PIAS3/pSTAT3/PD-L1 axis. This allows tumor cells to evade apoptosis, proliferation, invasion, migration, and the host immune system, resulting in the progression of gastric carcinoma [168]. BART-miRNA expression is notably high in NPC and gastric cancer, indicating that BART-miRNAs play a crucial role in the pathogenesis of EBV-related epithelial malignancies [171]. More information can be seen in Table 1.

#### 3.1.3 KSHV

KSHV, also known as HHV-8, is a herpes virus with a dsDNA genome ranging from 165 to 170 kb. It is a crucial contributor to the development of several types of cancers and severe and numerous malignancies in immunocompromised people. Multicentric castleman disease (MCD), primary effusion lymphoma (PEL), and KS are cancers caused by KSHV [172]. HHV-8 exploits various methods to improve cell survival and overcome anoikis, resulting in metastatic Kaposi's sarcoma [142]. vFLIP is a viral protein that is frequently expressed in tumor cells and has many functions, including increasing the expression of the inflammatory cytokines IL-8 and IL-6, initiating the NF-B cascade regulatory mechanism, controlling inflammation, cell proliferation, and the immune response. vFLIP expression activates the RelA/p65 nuclear transmitter and the classical NF-kβpathway in MVECs. Because vFLIP-mediated NF-kβ activation inhibits anoikis caused by MVEC cell separation, vFLIP serve a crucial role in NF-kβ activation. Through controlling and regulating COX-2/PGE2 expression, vFLIP is able to prevent the suppression of initial anoikis in primary endothelium cells, which is critical in regulating anoikis in many malignancies. In an NF-kβ dependent mechanism, v-FLIP/K13 viral oncoprotein causes tumorigenic effects by increasing COX-2 host protein and its inflammatory metabolite PGE2. COX-2 inhibits NF-kβinduction by v-FLIP/K13, leading to AR. vFLIP expression accelerates spindle cell differentiation and increases cytokine secretion by phosphorylating p38, FAK, RSK, Rac1-GTPase, Src, and Akt [173,174].

KSHV can activate FAK by causing the main endothelium to express vGPCR. vGPCR acts as a mediator; it can activate AP-1, ERK1/2, NF-kβ, induce migration, and inhibit anoikis. It's conceivable that vGPCR promotes EC survival by triggering the NF-B signaling pathway via the PI3K/Akt pathway. VGPCR can also activate MAPK-SAPK, a protein implicated in cell proliferation, angiogenesis, and inflammation, and also activate transcription factors such as AP-1, CREB, NFAT, and HIF1a, leading to VEGF production and angiogenesis. These factors can help cancer cells survive longer and contribute to the disease's development. It's conceivable that vGPCR promotes EC survival by activating the NF-kβ signaling pathway through the PI3K/Akt pathway [175].

#### 3.1.4 HPV

HPVs are uncoated dsDNA viruses wrapped in a 20-sided capsid with 72 capsules on each side. The size of their genome varies according to the kind of HPV. However, it is generally approximately 8000 kb. HPV contains two structural proteins, L1 and L2, which are efficient in infecting humans and causing virus infection [176,177]. Approximately 38% of all cancers related to the virus are caused by HPV and EBV.

These viruses' proteins can alter cancer cell behavior by suppressing tumor suppressor signaling, blocking apoptotic pathways, boosting AR, promoting metastasis, immunological inaccessibility, inflammation, and angiogenic stimulation [178]. Virus protein expression is related to host cell development and is required for viral genome replication in differentiated cells [179]. E6 and E7 are two proteins in HPV that play an important role in cancer formation. The E7 protein affects RB and acts via the destruction of this protein. RB interacts with the transcription factor E2F1, inhibiting its activity in the cell transcription machinery. E6 also interacts with two essential cellular regulatory proteins i.e., p53 and BAK34, resulting in cellular apoptosis resistance and decreased chromosomal stability. Furthermore, E6 and E7 proteins can interact with two tumor-inhibitory genes, BRCA1 and BRCA2 [180].

Erbin is a protein associated with the basolateral membrane. Erbin is a negative regulator of the Ras-Raf-ERK signaling cascade, producing phosphorylation, nuclear translocation, and STAT3 transcriptional activity in cervical cancer (CC) cells while organizing cell polarity and controlling cell adhesion. Anoikis in CC cells is clearly inhibited by overexpression of STAT3C and transcription of STAT3 by IL-6. IL-6 can activate STAT3 and boost erbin expression at the same time. The effects of IL-6 on STAT3 activation and AR are enhanced when the erbin gene is silenced. Erbin deficiency enhances the development and activation of metastasis in CC mice and AR in CC cells, both in vitro and in vivo. Erbin is a negative regulator of STAT3, and the IL-6/STAT3/Erbin loop is involved in CC growth and metastasis progression [181]. It has been observed that epithelial cells transformed with papillomavirus E6/E7 oncoproteins lead to AR, which is obtained via activating PKB/Akt, a crucial factor in cell transformation, by triggering Ras by Notch1 [182]. Anoikis is inhibited by the 600-kDa protein associated with retinoblastoma, or p600, and studies have revealed that the link between E7 BPV and p600 aids anoikis escape in cells separated from the cell matrix [183]. The transcriptional repression of E-cadherin is a crucial mechanism for its decrease during EMT. Several suppressors, including ZEB-1 and ZEB2, SIP1, Twist, Snail1, and Snail2, may bind to the box-E motif and reduce E-cadherin transcription. E6 and E7 have been shown to promote EMT by increasing the expression of EMT-activating transcription factors such as SLUG, TWIST, ZEB1/2, and SIX1 while decreasing the expression of E-cadherin [142,184,185]. Additionally, PTPN13 is a signaling molecule implicated in cell differentiation and the induction of carcinogenesis. Also, this protein plays a crucial role in cellular inhibition by interacting with the Fas receptor [186]. Microarrays are short non-coding RNAs that regulate gene expression and are involved in different cancer-related cellular processes [187]. As a result, any regulatory error in miR, as well as interactions between viral gene products and miR, can produce major regulatory changes in the cell, resulting in cancer phenotypes [13,142]. MiR-125 reduces the regulation of MMP-2, MMP-9, and N-cadherin. In contrast, E6/E7 proteins suppress the miR-125 expression, and as a result, viral oncoproteins can indirectly promote metastasis and tumor development by regulating cellular microarray expression levels. Oncoprotein E6 suppresses the p53 protein in CC cells, and the E6-E6AP complex binds to p53 and stimulates and promotes its destruction. In CC, the E6-p53 pathway has been related to altered miR-22 expression and its downstream target (HDAC6), which may play a role in the genesis and progression of CC [142,186,187]. Fatemipour et al. investigated the relationship between anoikis and human papillomavirus infection in prostate cancer cells [188]. In this study, cellular factors involved in apoptosis (BCL-2 and survivin), anoikis (SLUG, E-Cadherin, N-Cadherin, PTPN-13, and TWIST), and tumor inhibitory proteins (Rb and p53) were examined in prostate cancer cells. In addition, the control group statistically examined the association between the expression levels of the mentioned cellular factors with the presence of viruses and viral genes (E2, E6, and E7). According to the findings of this study, the expression levels of tumor inhibitory factors (Rb and p53) and apoptotic inhibitory proteins (BCL-2 and survivin) were significantly decreased in cancer tissues and virus-infected control selected tissues. In HPV-infected cancer cells, they also reported the expression levels of anoikis inhibitors (N-cadherin, SLUG, and TWIST) and anoikis stimulants (E-cadherin and PTPN13) increased and decreased, respectively. At the end of the study, the scientists suggested that HPV infection may produce cancer cell AR by altering the expression level of cellular genes involved in anoikis. This may eventually promote the spread and development of prostate cancer [188].

As mentioned above, AR is essential for metastasis. On another side, there is evidence demonstrating HPV aggravates the risk of cancer metastasis. As mentioned above, AR is essential for metastasis. On another side, there is evidence demonstrating HPV aggravates the risk of cancer metastasis. For example, it has been reported HPV oncoproteins stimulate EMT and metastasis by regulating β-catenin, JAK/STAT/SRC, RAS/MEK/ERK, and/or PI3k/Akt/mTOR signaling pathways [189]. Also, HPV-16 E6 may contribute to tumor invasion and metastasis by upregulating EGFR, AKT2, and CCND1 levels through negatively regulating miR-2861 expression in CC cells [190]. Therefore, there is a possibility that the virus can lead to the stimulation of metastasis by overcoming anoikis, however, more experimental studies should be done to understand this mechanism and gap.

## 4 Conclusion

Normal cell proliferation and tissue homeostasis are required for ECM cell transplants [191]. When these interactions are disrupted, a kind of cell death known as anoikis occurs, and AR is a key mediator in metastasis [4]. Metastasis is a multi-stage process that accounts for approximately 90% of cancer fatalities [192]. Chronic infections with carcinogenic viruses cause approximately 20% of all human cancers [15]. Carcinogenic viruses have a variety of effects on the path to AR, including the progression of carcinogenesis and metastasis in virus-associated malignancies [193]. They capture cellular proteins and various pathways implicated in vital physiological processes including metabolic reprogramming, activating integrin switching, activating survivin, and inactivating the apoptotic cascade pathway of the EMT process in order to maintain cell viability and thus save isolated cells. Directed towards anoikis from the extracellular matrix, resulting in malignancy development [51,194]. Carcinogenic viruses that induce AR appear to be a successful and effective strategy for maintaining cancer cells alive, migrating to other organs, and populating virus-infected cancer cells in new locations. As a result, healthy and normal cells can get infected, and viruses can easily spread throughout the body [72]. For example, Hepatitis B virus X protein and KSHV vFLIP control PAK1 and COX-2, allowing infected cancer cells to circumvent anoikis and prevent and inhibit metastasis in HCC and Kaposi's sarcoma, respectively [4]. HPV E7 can bind to the 600 kDa or p600 protein associated with retinoblastoma, thereby allowing isolated infected cells to survive in this condition [195]. Future research and studies aimed at better understanding the mechanisms of anoikis overcoming carcinogenic viruses in cancer could introduce new molecular targets for designing new therapeutic approaches [196].

With regard to the critical role of oncoviruses in cancer development, metastasis and potentially regulating AR, it is logical to think that the eradication of known risk factors related to lifestyle such as a reduction in risky sexual behaviors or screening blood-borne viruses can suppress the development of these malignancies, AR, and metastases; especially, since it has been revealed that oncoviruses co-infection such as coinfection HPV and EBV [[197], [198], [199]] or HBV/HIV and HCV/HIV [[200], [201], [202]] are able to play an essential role in the cancer progression. Meanwhile, prevention of oncovirus infection using the upcoming or existing vaccines, the development of effective antiviral drugs/vaccines, and paying more attention to the combination of anticancer drugs with antiviral drugs in the treatment of cancer patients infected with oncoviruses, might significantly decrease the rate of virus-associated malignancies and their progression to invasive forms that are responsible for most cancer-related deaths.

## Rights and permissions

Fig. 1 is fully adapted from a work by Qin et al. [73] published in the BMC journal of Hematology and Oncology, without applying any changes. All the rights are preserved for the authors and the paper is accessible through the link: https://rdcu.be/dqiHY. This article is licensed under a Creative Commons Attribution 4.0 International License, which is accessible through the following link: http://creativecommons.org/licenses/by/4.0/. The Creative Commons Public Domain Dedication waiver (http://creativecommons.o rg/publicdomain/zero/1.0/) applies to the data made available in this article.

## CRediT authorship contribution statement

**Zahra Sobhi Amjad:** Conceptualization, Data curation, Formal analysis, Funding acquisition, Investigation, Methodology, Project administration, Resources, Software, Supervision, Validation, Visualization, Writing – original draft, Writing – review & editing. **Ali Shojaeian:** Writing – review & editing. **Javid Sadri Nahand:** Investigation, Writing – review & editing. **Mobina Bayat:** Writing – review & editing. **Mohammad Taghizadieh:** Writing – review & editing. **Mosayeb Rostamian:** Project administration. **Farhad Babaei:** Investigation. **Mohsen Moghoofei:** Conceptualization, Data curation, Formal analysis, Funding acquisition, Investigation, Methodology, Project administration, Resources, Software, Supervision, Validation, Visualization, Writing – original draft, Writing – review & editing.

## Declaration of competing interest

The authors declare that they have no known competing financial interests or personal relationships that could have appeared to influence the work reported in this paper.
